# Social Interactions of Dat-Het Epi-Genotypes Differing for Maternal Origins: The Development of a New Preclinical Model of Socio-Sexual Apathy

**DOI:** 10.3390/biomedicines9070778

**Published:** 2021-07-05

**Authors:** Anna Brancato, Sara L. M. Lo Russo, Anna Sara Liberati, Cristiana Carbone, Silvia Zelli, Giovanni Laviola, Carla Cannizzaro, Walter Adriani

**Affiliations:** 1 Department of Health Promotion, Mother and Child Care, Internal Medicine and Medical Specialties of Excellence “G. D’Alessandro”, University of Palermo, 90127 Palermo, Italy; anna.brancato@unipa.it (A.B.); carla.cannizzaro@unipa.it (C.C.); 2 Center for Behavioral Sciences and Mental Health, Istituto Superiore di Sanità, 00161 Rome, Italy; lorusso93@icloud.com (S.L.M.L.R.); cricarbone@hotmail.it (C.C.); silvia.zelli@gmail.com (S.Z.); giovanni.laviola@iss.it (G.L.); 3 Faculty of Psychology, Università Telematica Internazionale “Uninettuno”, 00186 Rome, Italy; a.liberati1@students.uninettunouniversity.net

**Keywords:** dopamine transporter, socio-sexual reward, social behavior, parent-of-origin effect

## Abstract

Social interaction is essential for life but is impaired in many psychiatric disorders. We presently focus on rats with a truncated allele for dopamine transporter (DAT). Since heterozygous individuals possess only one non-mutant allele, epigenetic interactions may unmask latent genetic predispositions. Homogeneous “maternal” heterozygous offspring (termed MAT-HET) were born from dopamine-transporter knocked-out (DAT-KO) male rats and wild-type (WT) mothers; “mixed” heterozygous offspring (termed MIX-HET) were born from both DAT-heterozygous parents. Their social behavior was assessed by: partner-preference (PPT), social-preference (SPT) and elicited-preference (EPT) tests. During the PPT, focal MIX-HET and MAT-HET males had a choice between two WT females, one in estrous and the other not. In the SPT, they met as stimulus either a MIX-HET or a WT male. In the EPT, the preference of focal male WT rats towards either a MIX- or a MAT-HET stimulus was tested. MIX-HET focal males showed an abnormal behavior, seeming not interested in socializing either with a female in estrous or with another male if MIX-HET. Focal MAT-HET males, instead, were very attracted by the female in estrous, but totally ignored the MIX-HET male. We assessed the expression of noradrenaline transporter (NET) in prefrontal cortex, hippocampus and hypothalamus, finding differences between the two offspring. MIX-HETs’ hypothalamus and hippocampus showed less NET than MAT-HETs, while the latter, in turn, showed higher NET than WTs. These behavioral differences between heterozygous groups may be attributed to different maternal cares received. Results allow preclinical understanding of epigenetic factors involved in social-behavior abnormalities, typical of many psychiatric disorders.

## 1. Introduction

Social interaction is composed by complex behaviors, which are essential for life in many mammalian species. In humans, these interactions are impaired in several psychiatric disorders such as autism, schizophrenia, major depression and social anxiety disorder. Recent evidence highlighted how different neurotransmitters, including oxytocin, dopamine, noradrenaline, serotonine and β-endorphin, are recruited in social behavior and both dopaminergic and noradrenergic systems seem to be especially relevant to social drive [1]. A wide body of literature has demonstrated that external stimuli, such as stress, often result in significant changes in dopamine (DA) and noradrenaline (NE) concentrations in the social brain regions [2,3]. Indeed, DA neurons, located in the ventral tegmental area (VTA) and substantia nigra (SN), innervate brain regions relevant to social play and affiliative behavior, such as the prefrontal cortex, the hippocampus, the striatum and hypothalamus [4]. When subjects are in contact with social stimuli, emotional processes having a positive or negative valence involve DA neurons originating in VTA [5,6]. It was observed that, following exposure of a new stimulus rat to a same sex focal rat, the activity of the VTA neurons increases during the social interaction [7]. Accordingly, the optogenetic enhancement of phasic DA activity in VTA cell bodies increases social behavior (ibidem). These concordant results demonstrate that DAergic projections from VTA neurons are involved in social interaction.

In previous studies, we employed dopamine transporter knocked-out (DAT-KO) rats [8,9] to investigate the role of DA in the modulation of neuro-behavioral processes [10,11,12,13]. However, as far as social interaction is concerned, we believe that heterozygous (DAT-HET) rats could be more informative than full KOs [14,15]: in fact, DAT-HETs seem to exhibit a more evident asocial behavior in comparison with WT and even KO rats. Due to the fact that they are heterozygous, DAT-HETs possess only one functional allele for the DAT gene, and this specific allele may be more vulnerable to epigenetic effects. Therefore, in this study, we implemented two possible mating combinations to generate these heterozygous rats: the first one originated by classical breeding of both male and female DAT-HET subjects, leading to “mixed” offspring with an inactivated allele being either of paternal or of maternal origin (MIX-HET); a second group was obtained by breeding of KO male rats with WT female dams. This one was hence named “maternal” (MAT-HET [16]) because the functional DAT allele is always of maternal origin. These offspring received a full repertoire of maternal care (see [17]: “HETs with WT dam” correspond to present MAT-HET) unlike the MIX-HETs, whose mothers were heterozygous for DAT. Due to the decreased maternal behavior of these HET females, with respect to WT mothers, one source of variation between MIX- and MAT-HETs is obviously attributable to a different rearing style. Additionally, the atypical KO-WT breeding implies that MAT-HETs likely inherit unknown patterns of DNA methylation and histone acetylation, from their KO fathers. Our hypothesis is that HET offspring belonging to the two epi-genotypes would differ depending on crucial factors, among which being cared from either a WT or a HET dam, since HET mothers are less inclined to maternal care. In fact, as we already observed in a previous work [17], HET mothers show an evident reduction in taking care of their pups compared to WT mothers. More specifically, HET dams considerably prefer self-grooming (almost four-fold) rather than grooming or liking their pups. This maternal carelessness is not present in WT dams, who show of course some levels of self-grooming but do not neglect grooming their pups. Such kind of “biased” breeding presently allows to isolate the maternal epigenetic factors alone. A simpler approach might be to foster HET subjects to WT mothers and WT subjects to HET mothers, but in this case the maternal factors risk to interact with the pups’ own genotype [18].

Preliminary studies conducted on MAT vs. MIX-HET rats [16,17] also showed the presence of a greater overall locomotor activity in MAT-HETs, which are also more active than MIX-HETs during the first hour of facility lighting (resting time), thus suggesting the presence of sleep alterations. Using a methylphenidate challenge (methylphenidate 1 mg/kg), MAT-HET’s locomotory activity did not change in response to the lower dose, while the latter was already effective in MIX-HET and WT rats. Additionally, during the Porsolt forced Swim test, MAT-HET subjects showed an instable profile frequently alternating passive flotation and active escape behaviors. These data, if transferred from animals to man, could be translated in the alternation between states of despair and struggle which can be associated with an unstable emotional state. Our present study is focused on the sociality of MIX-HET and MAT-HET rats, to determinate if MIX-HET and MAT-HETs could become a new preclinical model of behavioral symptoms of mental disorders based on their epigenetic modification. Since in both groups the genotype is heterozygous and behavioral variations are, as already underlined, likely due to epigenetic impact resulting from their dam, the two are henceforward termed “epi-genotypes”. The two epi-genotypes, together with the WT comparison group, underwent three tasks: the partner preference (PPT), the social preference (SPT) and the elicited preference (EPT) tasks. These tests were chosen because they were considered functional for the targeted and comparative observation of the social behavior expressed by the three groups. Therefore, we adapted this kind of simple-choice test to our laboratory rats.

Our previous research [14] was based on encounters among all possible genotypes (WT, HET, KO, in a “3 × 3” design); presently, we tested instead offspring from HET mothers and HET fathers (MIX epi-genotype) versus WT mothers and KO fathers (MAT epi-genotype). In our own ethogram for scoring of the preference protocol, we had a total of eight behavioral items: one item serving position, three for social behaviors and four for non-social behaviors. In addition, we observed that social alterations in heterozygous DAT rats were associated with neurochemical alterations in norepinephrine neurotransmission, with increased levels of NE in the hippocampus and hypothalamus with respect to WT counterparts (ibidem), suggestive of a DA-NE crosstalk essential for the proper functioning of the social brain. DA and NE signaling are both essential to prefrontal and parietal attentive activities [19]. In addition, as it was suggested by early microdialysis studies [20,21,22], in brain regions where DAT expression is low or absent, such as prefrontal cortex and hippocampus, extracellular DA is effectively cleared by NE transporter [23,24], and this is likely to occur in DAT-KO subjects. On the other hand, in the striatum where DAT expression is abundant and NET-positive fibers show a sparse and heterogeneous distribution [25], the physiological significance of NET in controlling DA uptake seems to be not relevant [20,21,22,23].

As a matter of fact, it is not surprising that a reduced activity of DAT may lead to alterations in the levels of NET, due to compensatory adaptation in the NE levels, thus contributing to altered modulation of cerebral functions [26].

In the present study, we employed immunofluorescence to assess the expression of NET in the prefrontal cortex, hippocampus and hypothalamus, brain regions relevant to social behavior [4] where we observed altered NE levels in heterozygous DAT rats [14].

## 2. Methods

All experimental procedures have been approved by the ISS animal welfare survey board on behalf of Italian Ministry of Health (formal license 937/2018-PR for project D9997.61, delivered to W. Adriani; plus pending license application for project D9997.110, filed on 19 March 2019, and audited on March 2020; plus formal license 1008\2020-PR for project D9997.110; both delivered to W. Adriani). Procedures were carried out in close agreement with the directive of the European Community Council (2010/63/EEC) and with the Italian law guidelines. All efforts have made to minimize the suffering of animals and to use as few animals as possible, according to the 3Rs principle.

### 2.1. Subjects

The generation of Wistar-Han DAT knockout rats was previously described elsewhere [8]. The original colony was maintained in a heterozygous-heterozygous breeding fashion; the animals were intercrossed for >10 generations at Istituto Italiano di Tecnologia (IIT, Genoa, Italy). Some progenitors were shipped to Istituto Superiore di Sanità (ISS, Rome, Italy), where male DAT-KO rats were bred with Wistar-Han WT females (Charles River, Lecco, Italy), to obtain a new G0 of founder heterozygous subjects. Present subjects are G2 from our ISS colony. 

All experimental subjects were adult male rats (>120 days old; average weight 500 g) born from colony just described, in our facility. We tested a total of 24 male rats belonging to two different epi-genotypes (MIX-HET vs. MAT-HET). 

The first one was composed by offspring of HET father X HET mother while the second by offspring of KO father x WT mother; two siblings out of six per dam were used. All 24 rats were used for the partner and social preference tests, and as stimulus for the elicited preference test. Additionally another 7 adult heterozygous male rats, 18 WT males and 6 WT females, all hosted in the colony, were used. The 7 male HET and 7 of the male WT rats were used as stimuli for the social preference test (termed “HZ”) while the remaining 11 WT males were used as focals for the elicited preference task. The 24 main male subjects, all heterozygous but belonging to two epi-genotypes (i.e MIX offspring from HET mothers and KO fathers versus MAT offspring from WT mothers and KO fathers), underwent counter-balanced encounters with either free choice for various kind of stimulus rats or acting themselves as stimulus. For the experiment 1 (partner preference), a female in estrous and one not in estrous were used as stimuli; for experiment 2 (social preference) 7 WT and 7 HET males (thereafter termed “HZ”) were used as stimuli. Finally, for experiment 3 (elicited preference) the MAT and MIX-HETs, which were previously used as focal, acted as stimuli and consequently the non-previously stimuli WT rats acted instead as focal.

### 2.2. Apparatus

The experimental apparatus consisted of a plexiglass box with smooth walls and floor (70 × 30 × 35 cm), composed of two identical environments separated by a central partition containing a door, and yet distinguishable one another due to the different color of the walls: the walls on the long sides and in the center were grey whereas those on the short-end sides of the box were either black or white. The central door was always kept open. Inside each one of the two terminal chambers, close to the shorter walls’ corner, an aluminum cage (15 × 15 × 40 cm) has been positioned 3 cm higher than the floor. The stimulus rat was delicately placed inside one of the cages. Then, inside the “black wall” room, the following were placed: for experiment 1, the female not in estrous; for experiment 2 the chamber was left empty; for experiment 3, a MAT or a MIX-HET, depending on the case. Finally, in the “white wall” room we placed: for experiment 1 the female in estrous; for experiment 2 a WT or HET (“HZ”) stimulus; for experiment 3 a MIX- or a MAT-HET (in order to provide the WT rat with the opportunity of choice). The whole procedure (experimental meetings) was carefully videotaped during both experiments 1 and 2, while for experiment 3 automatic data were exploited.

### 2.3. Protocol

Partner, social and elicited preference tasks, spaced apart for at least one month, were each effectuated in a total of 4 consecutive days. The first two days were used for habituation and the other two were needed for testing. Habituation was conducted separately for focal and stimulus rats; during the first habituation day, in order to obtain stimulus rats used to be confined within the aluminum cage, we individually placed them inside of it for 15 min each; on the second day of habituation, all focal rats were placed for 30 min inside the apparatus where the two empty aluminum cages were located. Then, from the third to the fourth day, we ran the encounters, in order to measure the preference of focal rats towards stimuli.

During partner and social preference tasks both groups of heterozygous animals (MIX- and MAT-HETs) acted as focal subjects while in the elicited preference task they acted as stimuli. At the beginning of all encounters, we gently placed the stimulus rats inside the aluminum cages; immediately after (second step), we gently placed the focal rats in the starting room. In experiment 1, rats started from the “black wall” room (in whose cages there was the female non in estrous); for experiment 2, rats also started from “black wall” room, but in this case, the cages were empty. Finally, for experiment 3, both MAT- and MIX-HET rats which previously acted as focal, were, in this case, placed as stimuli in either chamber’s aluminum cage in a counterbalanced order. Instead, the focal WT rats (which were tested twice, on days 3 and 4) were placed once in the stimulus MAT-HET’s chamber and once in the MIX-HET’s. Both stimulus and focal rats were left there for a total of 15 min each. Focal rats, then, had the possibility to freely cross the door and enter in either chambers.

During the second day of testing (day 4), we again used the same focal subjects used to meet different stimulus rats in the previous experiment. This was in order to prevent final preference to be biased by one given stimulus in particular. Thus, male rats of different epi-genotypes met as stimulus some female rats, in both estrous and non-estrous conditions (exp 1) as well as different genotypes (exp 2: WT or HET male “HZ”) in a counterbalanced order. For experiment 3, the same MAT- and MIX-HET subjects, this time acting as a stimulus, were exchanged places (inside the aluminum cage) for days 3 and 4 in a counterbalanced order. The floors of each chamber and cage were thoroughly cleaned between animals with water and ethanol (2:1) and the test was carried out under red and dim white illumination.

An Observer XT 10 (NOLDUS, NL) was used in order to carefully analyze the videotapes obtained from experiment 1 and 2. We also created a behavioral ethogram in order to describe the interactions between focal and stimulus rats, totalizing 8 behavioral items.

We divided the ethogram first according to the two main positions of the rats (namely “inside” and “outside” the black and white wall chamber) and secondly to the two main groups of behaviors:

- Non-social behaviors: wall exploration, wall rearing, self-grooming, inactivity;
- Social behaviors: cage exploration, cage rearing, social sniffing.

For each behavior, we analyzed the total duration, total number (frequency) and the latency.

For experiment 3, we used Cage controller 1.27 for Dark light for Rat and Mouse (PRS, Rome, Italy), which allows us to score: (1) each subject’s motor activity (beam interruption per second) in either compartment; (2) time spent in each compartment (both forepaws and hind paws in a same compartment); (3) transitions (number of times a subject crosses the door between the two compartments). The compartment was divided into two sectors, one with two photocells close to the aluminum cage and the other one with two photocells close to the central door. This allowed us to obtain the above parameters separately for sectors “near to” and “far from” the cage.

Data were divided into 300 s intervals (bins).

### 2.4. Ex Vivo Net Immunofluorescence

The expression levels of noradrenaline transporter (NET) in WT, MIX-HET and MAT-HET rats were investigated by immunofluorescence in the prefrontal cortex (prelimbic and infralimbic sub-regions), hippocampus (dentate gyrus, CA1, CA2 and CA3 subregions) and hypothalamus (ventromedial and arcuate nuclei), by employing *n* = 6 rats per group.

All rats were given a lethal dose of 10% chloral hydrate i.p. and transcardially perfused with cold phosphate-buffered saline (PBS; pH 7.4) followed by fixation with cold 4% paraformaldehyde in PBS. Brains were dissected and post-fixed in the same fixative at 4 °C. Coronal sections were prepared on a vibratome at 35 μm thickness. Serial slices were collected through the rostral-caudal dimension of the brain (every 6th slice) and stored at 4 °C in 0.05% sodium azide in PBS until immunofluorescence processing. 

Immunofluorescence was performed as previously described [27], with a few modifications. Sections (six per animal) were washed in PBS for 30 min and incubated in blocking solution (3% normal goat serum (NGS), 0.3% Triton X-100 in PBS) for 2 h at room temperature under gentle shaking. Sections were then incubated in primary antibody solution for 24 h at 4 °C under gentle shaking (3% NGS, 0.3% Tween-20 in PBS, with anti-NET antibody, 1:200, Invitrogen, Waltham, MA, USA). Sections were washed in PBS for 1 h, incubated in secondary antibody for 2 h under gentle shaking (goat anti-mouse Alexa Fluor 594, 1:200; Jackson ImmunoResearch, West Grove, PA, USA). After 1 h washing in PBS, slices were briefly incubated with DAPI (1 mg/mL). Sections were slide mounted in Vectashield (Vector Laboratories, Burlingame, CA, USA) and cover slipped before imaging.

NET immunofluorescence was assessed in the prefrontal cortex (prelimbic and infralimbic sub-regions, from 3.2 to 2.7 mm from bregma), hippocampus (dentate gyrus, CA1, CA2 and CA3 subregions, from −3.14 to −3.6 mm from bregma) and dorsomedial hypothalamus (from −3.14 to −3.6 mm from bregma), according to [28], and the reported regional distribution of NET in the rat brain [25]. Images (one per section) were acquired on a Meiji Techno fluorescence microscope at 40× magnification, by employing Deltapix Insight imaging software (version 6.4.7, Smorum, Denmark). NET-p positive immunofluorescence was quantified by using ImageJ (NIH) and expressed as arbitrary units of integrated density values for each individual.

### 2.5. Data Analysis

Results of behavioral scoring were analyzed in experiments 1 and 2 using repeated measures analysis of variance (ANOVA). Our model was a 2 × 2 × 3 design: one between-subjects factor was the focal genotype (MIX-HET and MAT-HET) while the other factors were within-subject. One factor was constituted by stimulus features (phenotype for Exp 1: WT female in estrous vs. WT female not in estrous; genotype for exp 2: WT male vs. HET male “HZ”); the last one was time constituted by 3 partial bins of five minutes each. In experiment 3, we evaluated automatically collected data by ANOVA with a 2 × 2 × 3 design. All factors were within-subject: stimulus genotype (two levels: MIX-HET vs. MAT-HET), position of the focal rat in the environment (two levels: “near to” or “far from” the aluminum cage) and time (3 levels: three partial bins of 5 min each). In a second step of analysis, we run two separated ANOVAs, one focalized on data linked to the position “near” and the other one focalized on data linked to the position “far” (both ANOVAs thus had a 3 × 2 design). Multiple post hoc comparisons were performed with the Tukey’s HSD test.

Immunofluorescence data from each brain region were tested for normality and equal variances, and analyzed by using one-way ANOVA, considering epi-genotype as statistical factor, followed by Tukey’s post hoc test, when necessary.

Statistical analysis was performed using StatView II (Abacus Concepts, Berkeley, CA, USA) and Prism 8.2 (Graphpad Software Inc, San Diego, CA, USA). Data are expressed as mean ± SEM. Significance level was set at *p* ≤ 0.05, NS = not significant.

## 3. Results

As already described in the introduction of this article, the heterozygous rats used in our study were obtained from two different sources of breeding: the first originated by classical breeding of both male and female DAT-HET subjects, leading to “mixed” offspring with an inactivated allele being either of paternal or of maternal origin (MIX-HET); the second by breeding of KO male rats with WT female dams. This one was hence named “maternal” (MAT-HET) because the functional DAT allele is always of maternal origin. The latter received a full repertoire of maternal care. MIX-HETs, instead, did not receive appropriate attentions because their mothers are heterozygous for DAT and their maternal behavior is less careful than that of the WT mothers [17]. Therefore, one source of the observed behavioral variation between MIX- and MAT-HETs may be attributable to a different maternal rearing style. In order to evaluate their social behavior, the two epi-genotypes together with the WT comparison group underwent three tasks: the partner preference (PPT), the social preference (SPT) and the elicited preference (EPT) tasks, the results of which will be described below.

### 3.1. Partner Preference

#### 3.1.1. Inside the Chamber with the Female in Estrous

**Figure 1:**
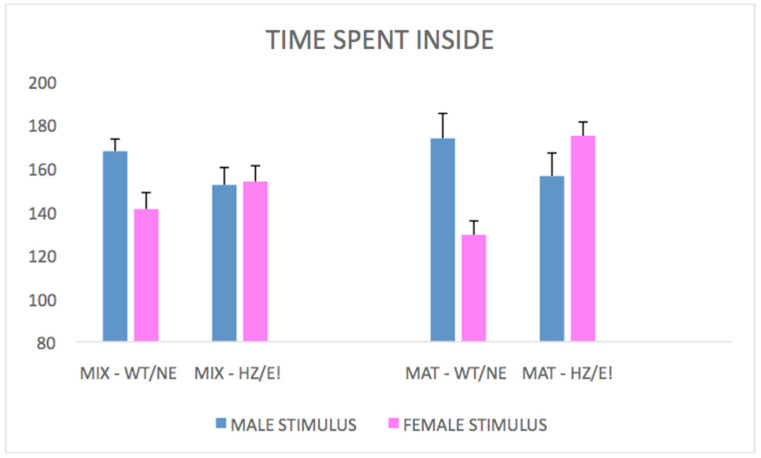
Total duration of time spent inside the chamber with a stimulus-conspecific subject (Bars represent performance of focal rats towards male stimuli, in Blue/exp. 1, or towards female stimuli, in Pink/ exp. 2). MIX-HET (left bars) focal male rats show a profile in which, by having as stimulus a female in estrous (E!) or a HET male (“HZ”), the total preference does not drastically change compared to having a female not in estrous (NE) or a WT male, respectively. MAT-HET focal male rats, instead, show a clear preference for the female in estrous (E!) but decreased preference for HET stimulus (HZ) rodents compared to male WT controls.

Total duration: analyzing the total duration time that males spent inside the room with target females, it was observed that MAT-HETs tended to spend more time in the chamber occupied by the female in estrous than in that with the female who was not in estrous (stimulus phenotypes * time, F (2,44) = 4.626; *p* = 0.0150). However, this finding was not equally observed in the MIX-HET epi-genotype (as indicated in Figure 1 as MIX-HZ) which, instead, did not seem particularly intrigued by the stimulus females.

Latency: post hoc analysis with Tukey resulted in HSD (44; K = 2) = 14.56). In the MAT-HET epi-genotype, the average latency was found to be lower when these males encountered the target female in estrous compared to the latency observed towards the non-estrous female (see Figure 1). In contrast, this effect was not observed in the MIX-HET epi-genotype. These data underline the clear preference and motivation of the MAT-HETs to reach the female in estrous, in contrast to the social apathy shown by the MIX-HETs (*p* < 0.05).

#### 3.1.2. Self-Grooming

**Figure 2:**
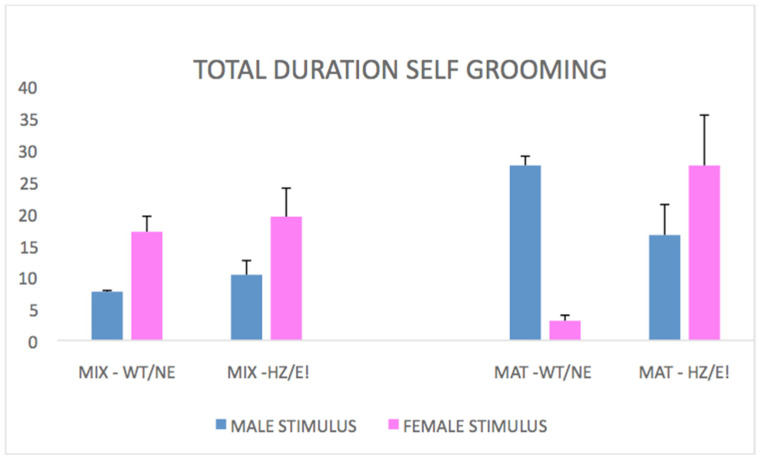
Total duration of self-grooming, whose profile is quite different for the two focal epi-genotypes (rats are the same as in Figure 1). In the focal rats of epi-genotype MIX-HET (left bars), the total duration of self-grooming is enhanced when the stimulus is a female. On the contrary, in the focal rats of epi-genotype MAT-HET (right bars), the total duration of self-grooming is enhanced when the stimulus is a female in estrous (E!) while it decreases with a HET stimulus (HZ).

Total duration: it was observed (Figure 2) that the MAT-HET epi-genotype was more involved in self-grooming behavior when in the chamber with the female in estrous, compared to what was shown with the female not in estrous. This behavioral profile was, instead, not shown at all by the MIX-HET epi-genotype, either with one or with the other female (stimulus phenotypes * focal genotypes, F (1,22) = 5.237; *p* = 0.0321).

#### 3.1.3. Wall Exploration

Total number: post hoc analysis with Tukey resulted in HDS (44; K = 2) = 1,12. As expected, the average frequency for “wall exploration” activity appeared to be statistically reduced when a male subject was placed in a room occupied by a female in estrous. However, this finding was mainly found in MAT-HET males, who seemed to be more interested in the female itself than in the environment (stimulus phenotypes F (1,22) = 5.737; *p* = 0.0256; stimulus phenotypes * time F (2,44) = 3.308; *p* = 0.459). Such profile was, instead, not found for the male MIX-HET epi-genotype, which did not seem to be particularly distracted by the receptive female.

#### 3.1.4. Wall Rearing

Total duration: post hoc analysis with Tukey resulted in HSD (44; K = 2) = 3.46. It was observed that the MIX-HET epi-genotype tended to spend more time rearing the wall rather than socializing with the female in estrous, while this exploratory behavior was totally absent in the MAT-HET epi-genotype which, however, was more focused on the female.

Total number: the average number of times that rats started rearing the wall was found to be greater within the chamber where the female in estrous was present (stimulus phenotypes, F (1,22) = 6.768; *p* = 0.0163). This phenomenon, however, was found to be valid only for MAT-HETs while it was absent in the MIX-HET epi-genotype.

Latency: it was observed that the average latency for wall rearing appeared to be higher when the subjects were placed inside the chamber with the female in estrous. Additionally, in this case, however, this result is applicable exclusively with regard to the MAT-HET epi-genotype, being totally absent in the MIX-HETs.

#### 3.1.5. Cage Exploration

**Figure 3:**
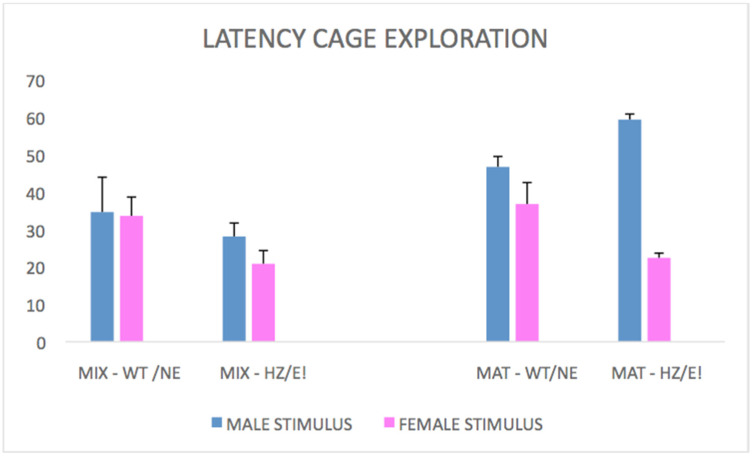
Latency to the exploration of the cage with stimuli (Rats are the same as in Figure 1). Within focal rats of epi-genotype MIX-HET (left bars), latency to exploration of the cage is quite similar across all stimuli and it is slightly reduced when the stimulus is a female in estrous. For the focal rats of epi-genotype MAT-HET (right bars), latency changes drastically depending on the stimulus: it is lower with the female in estrous (E!) and higher with a HET stimulus (HZ).

Latency: the latency for cage exploration (Figure 3) has gradually decreased in the MAT-HETs during their stay in chamber with the female in estrous (stimulus phenotypes F (1,22) = 4.862; *p* = 0.0382; stimulus phenotypes * time F (2,44) = 2.741; *p* = 0.0755). This data appear to be particularly interesting when compared to the MIX-HET epi-genotype, in which this phenomenon was found to be much less pronounced.

#### 3.1.6. Cage Rearing

Total duration: both MAT-HET and MIX-HET epi-genotypes spent more time in “CAGE REARING” when in the chamber with the female in estrous (as expected) (stimulus phenotypes F (1,22) = 7.980; *p* = 0.0099).

Latency: consistently with the other findings, the average latency for cage rearing (when in the chamber with the female in estrous) was found to be decreased in the MAT-HET epi-genotype and conversely increased in the MIX-HET epi-genotype (stimulus phenotypes * focal genotypes F (1,22) = 5.058; *p* = 0.0349; stimulus phenotypes * focal genotypes * time, F (2,44) = 4.045; *p* = 0.0244).

#### 3.1.7. Social Sniffing

Total duration: post hoc analysis with Tukey resulted in HSD (44; K = 2) = 4.24. When there was a female in estrous, total duration of social sniffing reduced in MIX-HETs (stimulus phenotypes F (1,22) = 7.581; *p* = 0.0116; stimulus phenotypes * time, F (2,44) = 3.308; *p* = 0.459). Although this profile was expected to be found, it nonetheless emphasizes no sex-related interest, compared to the MAT-HET epi-genotype.

#### 3.1.8. Inactivity

Total number: both MAT-HET and MIX-HETs showed, in a similar way between the two epi-genotypes, a gradual “inactivity” once placed into the chamber with the female in estrous (i.e., fragmented inactivity). The former because they were attracted to the female, the latter, on the contrary, because they were not interested.

### 3.2. Social Preference Test

#### 3.2.1. Inside the Chamber with a Male Stimulus

Total duration: as expected, focal MIX-HET epi-genotypes were observed to spend less time in the chamber together with a stimulus of the same genotype, compared to a WT stimulus (*p* < 0.05). This result (see Figure 1) appears even more pronounced in the MAT-HET epi-genotype (stimulus genotypes * focal genotypes, F (1,15) = 3453; *p* = 0.0829; stimulus genotypes * focal genotypes * time, F (2,30) = 860; *p* = 0.09422). Indeed, focal MAT-HETs spent much less time in the chamber with a MIX-HET (“HZ”) stimulus rat.

Total number: it was noted that the number of times in which a focal rat entered in the chamber with a “HZ” (MIX-HET STIMULUS) was statistically lower for a MAT-HET compared to a MIX-HET focal rat. While MIX-HETs entered a considerable number of times in the room where there was another MIX-HET stimulus, MAT-HETs entered in the chamber much lesser (nearly a −25%) (stimulus genotypes, F (1,13) = 3.539; *p* = 0.0825).

Latency: observations showed that in the MIX-HET epi-genotype, the average latency to enter a chamber with a stimulus of the same genotype was somewhat lower than for the WT control stimuli (Figure 2). Instead, this latency was found to be much higher (*p* < 0.05) for the MAT-HETs (stimulus genotypes * focal genotypes F (1,15) = 3.875; *p* = 0.0678; stimulus genotypes * time F (2,30) = 3.370; *p* = 0.0478; stimulus genotypes * focal genotypes * time, F (2,30) = 3.928; *p* = 0.305).

#### 3.2.2. Self-Grooming

Total duration: Tukey post hocs resulted in HDS (15; K = 2) = 3.375. Opposite results for the two epi-genotypes were noted (Figure 2): when in the chamber with a “HZ” MIX-HET stimulus, the average total duration of self-grooming in focal rats was observed to be greater for MIX-HETs and lower for MAT-HETs, in comparison to control WT stimuli observed in the same situation (*p* < 0.05).

Latency: when in the same chamber with a “HZ” MIX-HET stimulus, the average latency was lower for the focal MIX-HET and higher for the focal MAT-HET (*p* < 0.05) compared to control WT stimuli observed in the same situation. 

#### 3.2.3. Wall Exploration

Total duration: data demonstrate that the total duration of wall exploration was different between focal MIX-HET and MAT-HET rats during their encounter with a MIX-HET (“HZ”) stimulus. Additionally, the total duration of this behavior increased in focal MAT-HET rats than in WT control stimuli. It is interesting to note that this trend was not found at all in the MIX-HETs (stimulus genotypes * focal genotypes, F (1,15) = 5.011; *p* = 0.0408), which actually showed an asocial behavior.

Latency: The average latency for focal MAT-HET rats appeared to be greater when their stimulus was a MIX-HET compared to when the stimulus was a WT control rat (stimulus genotypes * focal genotypes F (1,15) = 3453; *p* = 0.0829). This trend has not shown at all in the focal MIX-HETs; the two epi-genotypes, therefore, differ considerably one another. This is reasonably due to having been cared by a DAT-HET dam versus a WT one.

#### 3.2.4. Wall Rearing

Latency: the average latency for wall rearing was lower for both MIX-HET and MAT-HET epi-genotypes when they were in contact with a MIX-HET stimulus (“HZ”) rat rather than a WT one (*p* < 0.05) (stimulus genotypes, F (1,15) = 4.562; *p* = 0.0496).

#### 3.2.5. Cage Exploration

Total number: data have shown that average frequency tend to decrease for the focal MAT-HET epi-genotype rats when they are placed in the same chamber with a stimulus, particularly if this is a MIX-HET (*p* < 0.05) (focal genotypes, F (1,15) = 5.001; *p* = 0.0410). MAT-HETs, therefore, appear to be asocial.

Latency: data have shown that the average latency is greater for the focal MIX-HET and lower for the focal MAT-HET (*p* < 0.05) when in contact with a MIX-HET (“HZ”), rather than with a WT control stimulus (stimulus genotypes * focal genotypes * time, F (2,30) = 3.593; *p* = 0.0399).

#### 3.2.6. Cage Rearing

Total number: post hoc analysis with Tukey yielded HSD = (30; K = 2) = 1.50. It was noted that the frequency for cage rearing has decreased in focal MAT-HET epi-genotype rats when in the same chamber with the “HZ” MIX-HET stimulus rat, confirming once again their asocial behavior.

#### 3.2.7. Social Sniffing

Latency: Data analysis has shown an emerging trend in the last bins (stimulus genotypes * focal genotypes * time, (2,30) = 3198; *p* = 0.0551). MAT-HET focal rats, when in contact with the “HZ” (MIX-HET) stimulus, showed a more pronounced latency for this behavior compared to the focal MIX-HET rats.

#### 3.2.8. Inactivity

Total duration and frequency: data have shown that MAT-HET focal rats start to be inactive more often (stimulus genotypes * focal genotypes, F (1,15) = 9.185; *p* = 0.084) and remain inactive for much more time when facing a MIX-HET stimulus (stimulus genotypes * focal genotypes, F (1,15) = 3.563; *p* = 0.0786). In other words, when focal MIX-HETs meet the MIX-HET stimulus, the frequency and total duration of “inactivity” appear lower (*p* < 0.05) compared to what observed with a WT control stimulus. Contrarily, the frequency and total duration of “inactivity” raise for focal MAT-HET rats when with MIX- HET stimuli. Once again, the MAT-HET epi-genotype was confirmed to be asocial. 

### 3.3. Elicited Preference Test

Through the analysis of the automatically collected data, we assessed whether there was a social preference for one of the two epi-genotypes (MAT-HET vs. MIX-HET males).

#### 3.3.1. Activity Rate and Transitions

ANOVA did not show significance for interaction of environment with epi-genotype of stimulus rats (stimulus genotype * environment-position * time, F (2,70) = 0.48; *p* = 0.9532) while interestingly a significant profile appeared for a number of transitions (F (2,70) = 5.989; *p* = 0.0040). Data indicate that there was a lower frequency of cage approaches compared to room entries towards the MAT-HET’s cage. Comparable entries and approaches emerged with a MIX-HET stimulus especially during the first partial bin. A reduction in the approach rate to the cage, when the time that the subjects spend in the sector containing that cage increases (see below), implies a longer average duration for each single approach: MAT-HETs seem to be the most attracted to it.

#### 3.3.2. Time Spent with Stimulus Rats

ANOVA for time spent in “sectors” within the chambers containing the stimulus rats did not show any significance (stimulus genotype * environment-position * time, F (2,68) = 2.535; *p* = 0.867). In presence of a strong effect of environment position (F (1,34) = 96.784; *p* < 0.001) separate ANOVA for the position “far” was carried out (stimulus genotype*time, F (2,70) = 1.59; *p* = 0.2096). Another ANOVA for the position “near” showed significance (F (2,70) = 4.96; *p* = 0.0231). Post hoc analyses and the threshold obtained with Tukey was 10.41 (df = 70; K = 3). Specifically, focal WT rats spent significant (*p* < 0.05) more time in the last partial bin (10–15 min) with the MAT-HET stimulus partner than with the MIX-HET one (Data not shown).

### 3.4. Ex Vivo Net Immunofluorescence

**Figure 4:**
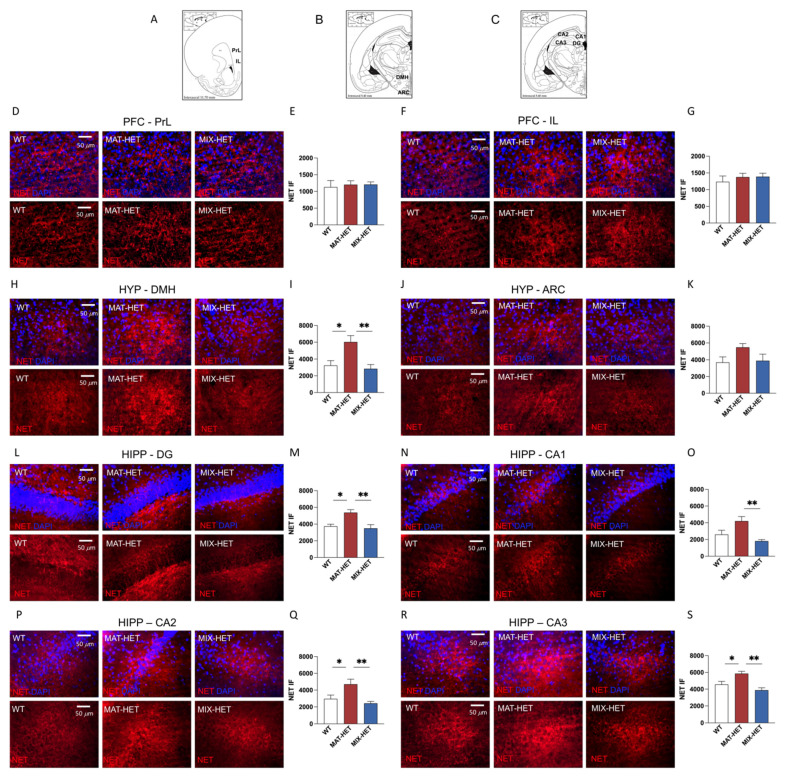
Comparison between MIX-HET and MAT-HET focal subjects in terms of NET immunofluorescence in the prefrontal cortex, hypothalamus and hippocampus. Images from NET-positive immunostaining were acquired at 40× magnification in the prelimbic (PrL) and infralimbic (IL) prefrontal cortex (**A**), dorsomedial (DMH) and arcuate (ARC) hypothalamus (**B**), dentate gyrus (DG), CA1, CA2 and CA3 subregions of the hippocampus (**C**). No differences in NET-positive immunofluorescence were observed in the subdivisions of the prefrontal cortex (**D**–**G**). MAT-HET rats displayed increased NET levels with respect to WT and MIX-HETs in the dorsomedial hypothalamus (**H**,**I**), while no differences were observed in the arcuate (**J**,**K**). Moreover, MAT-HET rats showed higher NET immunofluorescence than WT and/or MIX-HET rats in the DG (**L**,**M**), CA1 (**N**,**O**), CA2 (**P**,**Q**) and CA3 (**R**,**S**). Each bar represents the mean ± S.E.M. of n = 6 rats. * *p* < 0.05; ** *p* < 0.01.

NET-positive immunofluorescence was quantified in brain regions relevant to social behavior (Figure 4A–C). The results of one-way ANOVA showed no significant effect of the epi-genotype on NET immunofluorescence in both prelimbic (F (2,15) = 0.07783, *p* = 0.9255) and infralimbic (F (2,15) = 0.3277, *p* = 0.7256) prefrontal cortex (Figure 4D–G). On the other hand, data from NET immunofluorescence in the dorso-medial hypothalamus showed a significant effect of epi-genotype (F (2,15) = 7.806, *p* = 0.0047), with MAT-HET rats displaying a significant increase with respect to WTs (q = 4.485, df = 15, *p* = 0.0164), whereas MIX-HETs showed a significant decrease when compared to MAT-HET rats (q = 5.129, df = 15, *p* = 0.0066) (Figure 4H,I).

No significant differences were observed in the arcuate nucleus (F (2,15) =, respectively 0.2357, *p* = 0.1288) (Figure 4J,K). When NET immunofluorescence in the hippocampus was analyzed, one-way ANOVA revealed a significant effect of the epi-genotype in the dentate gyrus (F (2,15) = 8.439, *p* = 0.0035) with a significant increase in MAT-HET rats with respect to WTs (q = 4.634, df = 15, *p* = 0.0133), and a significant decrease in MIX-HET rats with respect to MAT-HETs (q = 5.352, df = 15, *p* = 0.0048) (Figure 4L,M). Moreover, the effect of epi-genotype was statistically significant in the CA1 region (F (2,15) = 7.619, *p* = 0.0052), where MIX-HET rats displayed a significant decrease with respect to MAT-HETs (q = 5.417, df = 15, *p* = 0.0044) (Figure 4N,O); in addition, we observed significant differences in the CA2 region (F (2,15) = 6.858, *p* = 0.0077) with a significant increase in MAT-HET epi-genotype with respect to WT rats (q = 3.837, df = 15, *p* = 0.0401) and a significant decrease in MIX-HET rats with respect to MAT-HETs (q = 5.006, df = 15, *p* = 0.0079) (Figure 4P,Q). Furthermore, one-way ANOVA revealed a significant effect of the epi-genotype in the CA3 region (F (2,15) = 10.58, *p* = 0.0014) and Tukey’s post hoc test highlighted a significant increase in MAT-HET rats with respect to WT rats (q = 4.209, df = 15, *p* = 0.0241) together with a significant decrease in MIX-HETs, when compared to MAT-HET rats (q = 6.401, df = 15, *p* = 0.0011) (Figure 4R,S).

## 4. Discussion

The MAT-HET epi-genotype exhibited a masked social profile only when in proximity to a WT stimulus rat. On the other hand, these focal rats behave similarly to a wild-type rat when in contact with a female in estrous. Specifically, MAT-HETs exhibited a higher “total duration” and frequency and a reduced “latency” for the exhibition of the social behaviors (cage exploration, cage rearing, social sniffing) toward the female in estrous while the contrary was displayed toward the “HZ”. This behavioral phenotype was associated with increased NET immunofluorescence in hippocampus and hypothalamus, with respect to WT and MIX-HET progeny. When acting as focal, MIX-HETs’ social profile appeared to be slightly different from that of a WT rat because it was not changing at all depending on available stimuli; it was simply lower when in contact with another HET male. These rats also exhibited a higher quantity of two non-social behaviors, such as “wall exploration” and “wall rearing”. Hence, a major interest for the environment was always shown, in addition to when they were in the same chamber with a female in estrous. Although MIX-HETs did not differ significantly in behavior from WT rats, they displayed decreased NET immunofluorescence in the hippocampus and hypothalamus with respect to MAT-HET rats, while the latter were greatly asocial only towards a same-sex “HZ”. Thus, because MIX-HETs display a behavioral profile which resembles that of non-mutant rats, the combination of external stimuli and epi-genotype can present itself as a promising basis to speculate that these rats could possibly become a behavioral model for socio-sexual apathy.

### 4.1. Detailed Summary of Data

After the analysis of all data, some key differences were highlighted in the behavior of focal MAT- vs. MIX-HET rats. Indeed, when they had a choice between various social stimuli, different results came out from the partner compared to the social preference test (SPP). While considering total duration spent inside the chamber allowing contact with a stimulus, focal male MIX-HETs appeared less interested toward a female in estrous compared to a female not in estrous. Thus, MIX-HET males are showing somewhat an altered sex-driven behavior. We noted that focal male MAT-HET rats tended to show a normal socio-sexual behavior, in that they were clearly more attracted by the female in estrous than by the one not in estrous. The opposite was true regarding time spent with a same-sex co-specific. Indeed, when compared to focal MIX-HET male rats, MAT-HET males tended to spend slightly less time in the chamber with a MIX-HET stimulus (HZ) compared to control WT stimulus rats. Therefore, rats with the same “MIXED” epi-genotype exhibited a marked reduction in general social interest.

Regarding the latency to enter a room, the MAT-HET epi-genotype quickly reached a female in estrous. On the other hand, when faced with a same-sex MIX-HET stimulus (HZ), MAT-HET males seem to lose any interest. In fact, focal MIX-HET epi-genotypes show similar latency to social interact regardless of type of genotype or sex of the stimulus. Indeed they showed comparable levels with a MIX-HET male and a WT male, or with a female in estrous or one not in estrous. This confirms the extremely different socio-sexual approach behavior shown by either MIX- or MAT-HET epi-genotypes.

Regarding self-grooming, opposite results in the performance of the two epi-genotypes were noted. Total duration of self-grooming by MAT-HET epi-genotype focal rats was greater when in the chamber with a female in estrous than with a female not in estrous. On the contrary, self-grooming activities were lower when in contact with the MIX-HET male stimulus, compared to WT male controls. Opposite results were registered for focal MIX-HET males, which seemed to be more stressed than focal MAT-HET ones when in contact with a female (regardless her hormonal condition), compared to when in proximity to a male stimulus.

Regarding the dynamics of chamber wall-and-cage exploration, it is noteworthy that both duration and frequency was reduced for the former, and increased for the latter, respectively, when the focal rats showed interest for the stimulus (namely, by sniffing the stimulus rat itself or its aluminum cage). The finding of a significant reduction in the interest for the chamber-wall was offered when a male MAT-HET focal rat was in the chamber with the female in estrous. On the other hand, this profile was absent for the MIX-HET epi-genotype denoting an abnormal behavior: in fact these males completely ignored sex triggers and displayed a peculiar “wall preference”.

Instead, when the stimulus was represented by a MIX-HET rat (“HZ”) compared to a WT control, the dynamics of the behavior was different. In fact, the interest shown for this subject by the MIX-HET focal male was greater, while it was much lower for the MAT-HET focal male. Regarding cage rearing, we noted that the average total number decreased for the focal MAT-HET rats (both if they have a female in estrous compared to control one, or a MIX-HET stimulus “HZ” compared to WT controls). MIX-HETs (characterized by having been reared by a heterozygous dam) exhibited strong tendencies to not discriminate among diverse stimuli as adults. On the contrary, the MAT-HETs (all heterozygous subjects reared by a WT dam) were showing overall overt climbing toward the receptive partner but never tried reaching any other heterozygous males. Namely, specifically exclusive interest for mating and no interest in same-sex social behavior was shown at all. In the elicited preference test, a slight but significant preference was shown by focal WT male rats, for the MAT-HET male stimulus. These behaviors indirectly provide indications that the MIX-HET subjects are somewhat generically avoided, above all in the last partial bins of a session.

When NET immunofluorescence was analyzed in brain regions relevant to social behavior, our data showed no significant difference in the prefrontal cortex amongst the three epi-genotypes. On the other hand, MAT-HET rats showed increased NET levels in discrete hippocampal subregions with respect to WT rats. In addition, MAT-HETs showed significant higher level in NET-positive immunofluorescence with respect to MIX-HET rats in the four subregions considered. A similar picture emerged from data analysis of NET immunofluorescence in the hypothalamus, where MAT-HET rats showed increased NET levels, with respect to both WT and MIX-HET rats.

### 4.2. Interpretation of Data

DAT-KO rats have been reported to exhibit some positive symptoms of schizophrenia such as psychomotor agitation, stereotypy, deficits of prepulse inhibition (PPI) and cognitive impairment [29]. Furthermore Rodriguiz et al. [30] reported that DAT-KO mice exhibit a marked aggressive phenotype. DAT-KO rats often engage in stereotyped and perseverative social behaviors which become more robust over time. These findings suggest that stereotypy and perseveration could distort the social interactions of KO rats. As far as their behavioral repertoires become restricted and inflexible, KOs show dominant traits. Thus far, nothing is known about the sociality of heterozygous subjects. Yet, social behavior is highly rewarding, thus insights can be drawn from literature; both DA and NE are involved in social behavior.

In detail, while DA modulates the rewarding and motivational aspect of social interaction, noradrenergic systems is involved in the attentional processes relevant to social behavior [31]. Interestingly, recent evidence shows that DA is strongly associated with motivation for social play whilst NE enhancement negatively modulates both motivation and expression of social play [32]. Sociality could be therefore influenced as well by quality of received maternal care during development. In a recent study [33], rats that received high levels of licking and grooming by their mothers spent significantly more time in social contact as adults with unfamiliar individuals, compared to subjects that received fewer levels of maternal care. In adult rats which received more maternal care, a reduced level of anxiety is linked with their greater social interaction. In contrast, another study, which has focused on the C57BL/6J strain, affirmed that rats receiving lower doses of licking and grooming from their mothers play more frequently and exhibit a more pronounced social behavior, compared to those who receive higher doses of licking and grooming [34].

Based on these observations, we aimed to compare brain levels of NET in MAT, MIX and WT subjects in order to evaluate their possible involvement in the behavioral abnormalities which we found in MATs. Indeed, mammalian social recognition rely on signals passed between individuals conveying information including sex, reproductive status, individual identity, ownership, competitive ability and health status, to modulate a variety of behaviors that are crucial for reproductive success, such as parent–offspring attachment, mate choice and territorial marking [35]. Indeed, social recognition is contingent on somatosensory stimulation, which evokes increased NE release from the brainstem to the vomeronasal pathway. This is a relatively direct route by which chemosensory stimuli mediate adaptive responses, in terms of endocrine state and behavioral responses through projections to the cortico-limbic brain regions, such as prefrontal cortex and hippocampus, and the hypothalamus [36,37,38,39].

The ex vivo analysis performed in the different heterozygous DAT epigenotypes, have clearly shown that NET-positive terminals in the hippocampal subregions (i.e., dentate gyrus (DG) and subregions (CA1, CA2 and CA3) of MAT rats are equal to almost the double than levels found in WT and MIX specimens, in the same brain regions.

Previous reports show that NE uptake activity in discrete brain regions is an important mechanism by which social recognition responses occur [40,41]. In accordance, the present evidence expanded our previous reports showing that HET rats displayed increased levels of NE in the hippocampus and hypothalamus, along with increased inactivity in the face of the social stimulus [14]. In particular, the immunofluorescent experiment confirmed a significant alteration in the noradrenergic transmission to the hippocampus and hypothalamus in HET rats, with epi genotype-specific differences of opposite type. These may underlie the different behavioral phenotypes of the two groups. Although only a tendency with respect to WT, MIX-HET male progeny showed a lower expression of NET in both hypothalamus and hippocampus with respect to MAT-HET rats. This evidence is consistent with the elevated level of noradrenaline measured in the same brain regions in similarly raised MAT-HET rats, reported previously (ibidem). On the other hand, MAT-HET offspring displayed marked increment in the hypothalamus and hippocampus of NET immune-fluorescence, compared to WT and MIX-HET rats. Despite the fact that a comprehensive comparative analysis of the hippocampal circuits and mechanisms underlying the wide range of social interactions explored in the present experiments (including social recognition to compare the co-specific male/male, male/female and cross-epi-genotype recognition), has not been reported yet, previous work has established that the hippocampal CA2 and CA3 regions are involved in social processing. Indeed, the genetic lesion of CA2 impaired social recognition [42], CA3 pyramidal cell plasticity and transmission contribute to the encoding of social stimuli [43]. Our data further suggest that altered noradrenergic transmission in DG may contribute to the wide range of social alteration observed in HET rats.

Therefore, it could be hypothesized that the socially anomalous behavior of MAT individuals, which are heterozygous for DAT (KO allele inherited from the father) and had been raised from a WT mother, compared to MIX subjects, which are heterozygous for DAT (from a heterozygous DAT-HET female), may be accounted by a high concentration of NE in brain regions relevant to social behavior. By this framework, it is possible to associate the concomitant NE hyperfunction and vice versa, the hypofunctionality of the DAT gene. In this regard, the evaluation of NE signaling in brain regions where NE increase is particularly relevant to socio-sexual behavior, such as the olfactory bulb, deserves further investigation in future studies.

### 4.3. Concluding Hypotheses about Dat-Het Epi-Genotypes

As a whole, MIX-HET male rats showed an altered social behavior: with the specifier of social apathy. This profile has been here associated to increased noradrenaline input to the hippocampal and hypothalamic regions. In EPT, we observed that WT rodents prefer to spend time with a MAT-HET stimulus. This can well be due to the fact that MIX-HET rats are rather apathic while WT controls are more easily compared to a MAT-HET rat. The latter, however, show an “almost bipolar-like” profile (see also [16]), ranging from no interest for a “HZ” male to a great excitation and approach toward a receptive female. MAT-HETs result attractive compared to MIX-HET stimulus rodents; also, MIX-HET male rodents seem more stressed (compared to MAT-HET ones) when in contact with a female, regardless her hormonal condition.

PPT was originally developed in Dr. Sue Carter’s laboratory [44] and assesses the extent of social contact and time in proximity to a partner relative to a stranger. In fact it has been used extensively to assess how different manipulations alter formation and maintenance of preferences for a mate in monogamous prairie voles and to a lesser degree in other monogamous species [45,46]. PPT is also commonly used to assess factors affecting social preferences for same-sex peers in meadow and prairie voles [47,48,49], and occasionally other rodents (e.g., [50]). A crucial factor seems to be represented by the fact that MIX-HET subjects had been raised by heterozygous mothers (as it is always the case in a classical colony setting). Our previous study [20] included observations demonstrating that MIX-HET mothers are much involved in self-grooming and/or to dig away from the nest, rather than caring for the pups. Instead, WT dams provided appropriate levels of licking and grooming to the pups: we proposed that, because of the style of maternal care received, WT female pups did not differ so much from MAT-HET pups [17], while MIX-HET females developed an altered circadian cycle. In that study, due to colony HET/HET breeding, we acknowledged that both halves of MIX-HETs were comprised (one comparable to MAT-HET and one not, as including DAT functional alleles randomly of paternal origin: this factor was obviously dropped here, by using KO fathers). As mother–infant interaction is known to modulate noradrenergic neurotransmission in the pups [51], we could not exclude the possibility that, for some (up to a half) of those DAT-HET females, the inactivated DAT maternal allele played a role. However, this is still under our investigation [18].

Moreover, another source of variability is that only half of MIX-HET subjects, born from a HET/HET breeding, will have functional maternal and mutated paternal alleles, while the other half of the siblings will have functional paternal and mutated maternal alleles. These limitations can be partly circumvented: breeding KO males with HET females will simply produce a half of MIX-HET offspring which will be entirely comparable to MAT-HET subjects, apart from the mother’s genotype (and cares). Maternal care has been shown to be of particular importance for the development of mammalian infants and their social behavior. Individuals that do not receive regular levels of licking and grooming by their mothers have been reported to exhibit higher levels of anxiety-related behaviors and reduced social motivation at adulthood. This “maternal effect” could explain at least partially the difference in the social behavior of the two epi-genotypes.

In a future study, we would like to evaluate the behavior of a next generation from present subjects: if female behavior is affected by her dam’s behavior [17], a carryover consequence may be hypothesized to pups which are in turn offspring from these two epi-genotypes. We are planning to cross-breed the two subtypes so that all parents will be of a same heterozygous genotype, yet the grandparents will differ.

## Data Availability

Data for these experiments are stored on a computer in the office of the corresponding author; such raw data can be made available upon request.

## References

[1] Pearce E., Wlodarski R., Machin A., Dunbar R.I.M. (2017). Variation in the β-endorphin, oxytocin, and dopamine receptor genes is associated with different dimensions of human sociality. Proc. Natl. Acad. Sci. USA.

[2] Gutknecht L., Popp S., Waider J., Sommerlandt F.M.J., Göppner C., Post A., Reif A., Hove D.V.D., Strekalova T., Schmitt A. (2015). Interaction of brain 5-HT synthesis deficiency, chronic stress and sex differentially impact emotional behavior in Tph2 knockout mice. Psychopharmacology.

[3] Strekalova T., Svirin E., Waider J., Gorlova A., Cespuglio R., Kalueff A., Pomytkin I., Schmitt-Boeher A.G., Lesch K.P., Anthony D.C. (2020). Altered behavior, dopamine and norepinephrine regualtion in stressed mice heterozygous in TPH2 gene. Prog. Neuropsychopharmacol. Biol. Psychiatry.

[4] Lopes P.C., König B. (2020). Wild mice with different social network sizes vary in brain gene expression. BMC Genom..

[5] Maniaci G., Picone F., van Holst R.J., Bolloni C., Scardina S., Cannizzaro C. (2017). Alterations in the Emotional Regulation Process in Gambling Addiction: The Role of Anger and Alexithymia. J. Gambl. Stud..

[6] Plescia F., Brancato A., Venniro M., Maniaci G., Cannizzaro E., Sutera F.M., De Caro V., Giannola L.I., Cannizzaro C. (2015). Acetaldehyde self-administration by a two-bottle choice paradigm: Consequences on emotional reactivity, spatial learning, and memory. Alcohol.

[7] Gunaydin L.A., Grosenick L., Finkelstein J.C., Kauvar I.V., Fenno L.E., Adhikari A., Lammel S., Mirzabekov J.J., Airan R.D., Zalocusky K.A. (2014). Natural neural projection dynamics underlying social behavior. Cell.

[8] Leo D., Sukhanov I., Zoratto F., Illiano P., Caffino L., Sanna F., Messa G., Emanuele M., Esposito A., Dorofeikova M. (2018). Pronounced Hyperactivity, Cognitive Dysfunctions, and BDNF Dysregulation in Dopamine Transporter Knock-out Rats. J. Neurosci..

[9] Cinque S., Zoratto F., Poleggi A., Leo D., Cerniglia L., Cimino S., Tambelli R., Alleva E., Gainetdinov R., Laviola G. (2018). Behavioral Phenotyping of Dopamine Transporter Knockout Rats: Compulsive Traits, Motor Stereotypies, and Anhedonia. Front. Psychiatry.

[10] Giros B., Jaber M., Jones S., Wightman R.M., Caron M.G. (1996). Hyperlocomotion and indifference to cocaine and amphetamine in mice lacking the dopamine transporter. Nature.

[11] Sora I., Wichems C., Takahashi N., Li X.-F., Zeng Z., Revay R., Lesch K.-P., Murphy D.L., Uhl G.R. (1998). Cocaine reward models: Conditioned place preference can be established in dopamine- and in serotonin-transporter knockout mice. Proc. Natl. Acad. Sci. USA.

[12] Gainetdinov R., Bohn L., Walker J.K., Laporte A.S., Macrae A.D., Caron M.G., Lefkowitz R.J., Premont R. (1999). Muscarinic Supersensitivity and Impaired Receptor Desensitization in G Protein–Coupled Receptor Kinase 5–Deficient Mice. Neuron.

[13] Spielewoy C., Roubert C., Hamon M., Nosten-Bertrand M., Betancur C., Giros B. (2000). Behavioural disturbances associated with hyperdopaminergia in dopamine-transporter knockout mice. Behav. Pharmacol..

[14] Adinolfi A., Zelli S., Leo D., Carbone C., Mus L., Illiano P., Alleva E., Gainetdinov R.R., Adriani W. (2019). Behavioral characterization of DAT-KO rats and evidence of asocial-like phenotypes in DAT-HET rats: The potential involvement of norepinephrine system. Behav. Brain Res..

[15] Sanna F., Bratzu J., Serra M.P., Leo D., Quartu M., Boi M., Espinoza S., Gainetdinov R.R., Melis M.R., Argiolas A. (2020). Altered Sexual Behavior in Dopamine Transporter (DAT) Knockout Male Rats: A Behavioral, Neurochemical and Intracerebral Microdialysis Study. Front. Behav. Neurosci..

[16] Carbone C., Brancato A., Adinolfi A., Lo Russo S.L.M., Alleva E., Cannizzaro C., Adriani W. (2020). Motor transitions’ peculiarity of heterozigous DAT rats when offspring of an un-conventional KOxWT mating. Neuroscience.

[17] Mariano S., Pardo M., Buccheri C., Illiano P., Adinolfi A., Russo S.L.M.L., Alleva E., Carbone C., Adriani W. (2020). Own or dam’s genotype? Classical colony breeding may bias spontaneous and stress-challenged activity in DAT-mutant rats. Dev. Psychobiol..

[18] Oggiano M., Buccheri C., Alleva E., Adriani W. (2021). Dopaminergic modulation of the circadian activity and sociability: Dissecting parental inheritance versus maternal styles as determinants of epigenetic influence. Behav. Brain Res..

[19] Arnsten A.F. (2004). Adrenergic targets for the treatment of cognitive deficits in schizophrenia. Psychopharmacology.

[20] Carboni E., Tanda G.L., Frau R., Di Chiara G. (1990). Blockade of the noradrenaline carrier increases extracellular dopamine concentrations in the prefrontal cortex: Evidence that dopamine is taken up in vivo by noradrenergic terminals. J. Neurochem..

[21] Di Chiara G., Tanda G.L., Frau R., Carboni E. (1992). Heterologous mono- amine reuptake: Lack of transmitter specificity of neuron-specific carriers. Neurochem. Int..

[22] Yamamoto B.K., Novotney S. (1998). Regulation of extracellular dopamine by the norepinephrine transporter. J. Neurochem..

[23] Morón J.A., Brockington A., Wise R.A., Rocha B.A., Hope B. (2002). Dopamine uptake through the norepinephrine transporter in brain regions with low levels of the dopamine transporter: Evidence from knock-out mouse lines. J. Neurosci..

[24] Borgkvist A., Malmlöf T., Feltmann K., Lindskog M., Schilström B. (2012). Dopamine in the hippocampus is cleared by the norepinephrine transporter. Int. J. Neuropsychopharmacol..

[25] Schroeter S., Apparsundaram S., Wiley R.G., Miner L.H., Sesack S.R., Blakely R.D. (2000). Immunolocalization of the cocaine- and antidepressant-sensitive l-norepinephrine transporter. J. Comp. Neurol..

[26] Ventura R., Morrone C., Puglisi-Allegra S. (2007). Prefrontal/accumbal catecholamine system determines motivational salience attribution to both reward- and aversion-related stimuli. Proc. Natl. Acad. Sci. USA.

[27] Brancato A., Castelli V., Lavanco G., Marino R.A.M., Cannizzaro C. (2020). In utero Δ9-tetrahydrocannabinol exposure confers vulnerability towards cognitive impairments and alcohol drinking in the adolescent offspring: Is there a role for neuropeptide Y?. J. Psychopharmacol..

[28] Paxinos G., Watson C. (1998). The Rat Brain in Stereotaxic Coordinates.

[29] Wong P., Sze Y., Chang C.C., Lee J., Zhang X. (2015). Pregnenolone sulfate normalizes schizophrenia-like behaviors in dopamine transporter knock-out mice through the AKT/GSK3β pathway. Transl. Phsychiatry.

[30] Rodriguiz R.M., Chu R., Caron M.G., Wetsel W.C. (2004). Aberrant responses in social interaction of dopamine transporter knockout mice. Behav. Brain Res..

[31] Vanderschuren L.J., Niesink R.J., Van Ree J.M. (1997). The neurobiology of social play behavior in rats. Neurosci. Biobehav. Rev..

[32] Achterberg E.J., van Kerkhof L.W., Servadio M., van Swieten M.M., Houwing D.J., Aalderink M., Driel N.V., Trezza V., Vanderschuren L.J. (2016). Contrasting Roles of Dopamine and Noradrenaline in the Motivational Properties of Social Play Behavior in Rats. Neuropsychopharmacology.

[33] Starr-Phillips E.J., Beery A.K. (2014). Natural variation in maternal care shapes adult social behavior in rats. Dev. Psychobiol..

[34] Franks B., Champagne F.A., Curley J.P. (2015). Postnatal maternal care predicts divergent weaning strategies and the development of social behavior. Dev. Psychobiol..

[35] Brennan P.A., Kendrick K.M. (2006). Mammalian social odours: Attraction and individual recognition. Philos. Trans. R. Soc. Lond. B Biol. Sci..

[36] Li C.S., Kaba H., Saito H., Seto K. (1989). Excitatory influence of the accessory olfactory bulb on tuberoinfundibular arcuate neurons of female mice and its modulation by oestrogen. Neuroscience.

[37] Li C.S., Kaba H., Saito H., Seto K. (1990). Neural mechanisms underlying the action of primer pheromones in mice. Neuroscience.

[38] Li C.S., Kaba H., Saito H., Seto K. (1992). Cholecystokinin: Critical role in mediating olfactory influences on reproduction. Neuroscience.

[39] Zhang X., Meeks J.P. (2020). Paradoxically Sparse Chemosensory Tuning in Broadly Integrating External Granule Cells in the Mouse Accessory Olfactory Bulb. J. Neurosci..

[40] Shang Y., Dluzen E.D. (2001). Nisoxetine infusion into the olfactory bulb enhances the capacity for male rats to identify conspecifics. Neuroscience.

[41] Zinn C.G., Bühler L., Cavalcante L.E., Schmidt S.D., Ferreira F.F., Zanini M.L., Furini C.R.G., de Carvalho Myskiw J., Izquierdo I. (2018). Methylphenidate induces state-dependency of social recognition learning: Central components. Neurobiol. Learn. Mem..

[42] Hitti F.L., Siegelbaum S.A. (2014). The hippocampal CA2 region is essential for social memory. Nature.

[43] Chiang M.C., Huang A.J.Y., Wintzer M.E., Ohshima T., McHugh T.J. (2018). A role for CA3 in social recognition memory. Behav. Brain Res..

[44] Williams J.R., Catania K.C., Carter C.S. (1992). Development of partner preferences in female prairie voles (Microtus ochrogaster): The role of social and sexual experience. Horm. Behav..

[45] Ahern T.H., Modi M.E., Burkett J.P., Young L.J. (2009). Evaluation of two automated metrics for analyzing partner preference tests. J. Neurosci. Methods.

[46] Kingsbury M.A., Goodson J.L. (2014). Pair bond formation is impaired by VPAC receptor antagonism in the socially monogamous zebra finch. Behav. Brain Res..

[47] Beery A.K., Routman D.M., Zucker I. (2010). Same-sex social behavior in meadow voles: Multiple and rapid formation of attachments. Physiol. Behav..

[48] Beery A.K., McEwen L.M., MacIsaac J.L., Francis D.D., Kob M.S. (2016). Natural variation in maternal care and cross-tissue patterns of oxytocin receptor gene methylation in rats. Horm. Behav..

[49] Anacker A.M.J., Christensen J.D., LaFlamme E.M., Grunberg D.M., Beery A.K. (2016). Septal oxytocin administration impairs peer affiliation via V1a receptors in female meadow voles. Psychoneuroendocrinology.

[50] Triana-Del Rio R., Montero-Domínguez F., Cibrian-Llanderal T., Tecamachaltzi-Silvaran M.B., Garcia L.I., Manzo J., Hernandez M.E., Coria-Avila G.A. (2014). Same-sex cohabitation under the effects of quinpirole induces a conditioned socio-sexual partner preference in males, but not in female rats. Pharmacol. Biochem. Behav..

[51] Kalpachidou T., Raftogianni A., Melissa P., Kollia A.M., Stylianopoulou F., Stamatakis A. (2016). Effects of a Neonatal Experience Involving Reward Through Maternal Contact on the Noradrenergic System of the Rat Prefrontal Cortex. Cereb. Cortex.

